# A Novel Strategy to Engineer Pre-Vascularized Full-Length Dental Pulp-like Tissue Constructs

**DOI:** 10.1038/s41598-017-02532-3

**Published:** 2017-06-12

**Authors:** Avathamsa Athirasala, Fernanda Lins, Anthony Tahayeri, Monica Hinds, Anthony J. Smith, Christine Sedgley, Jack Ferracane, Luiz E. Bertassoni

**Affiliations:** 1 Division of Biomaterials and Biomechanics, Department of Restorative Dentistry, School of Dentistry, Oregon Health and Science University, Portland, OR USA; 2 Department of Biomedical Engineering, School of Medicine, Oregon Health and Science University, Portland, OR USA; 3 School of Dentistry, University of Birmingham, Birmingham, UK; 4 Department of Endodontology, School of Dentistry, Oregon Health and Science University, Portland, OR USA; 5 Center for Regenerative Medicine, Oregon Health and Science University, Portland, OR USA; 6 Bioengineering Laboratory, Faculty of Dentistry, University of Sydney, Sydney, NSW Australia

## Abstract

The requirement for immediate vascularization of engineered dental pulp poses a major hurdle towards successful implementation of pulp regeneration as an effective therapeutic strategy for root canal therapy, especially in adult teeth. Here, we demonstrate a novel strategy to engineer pre-vascularized, cell-laden hydrogel pulp-like tissue constructs in full-length root canals for dental pulp regeneration. We utilized gelatin methacryloyl (GelMA) hydrogels with tunable physical and mechanical properties to determine the microenvironmental conditions (microstructure, degradation, swelling and elastic modulus) that enhanced viability, spreading and proliferation of encapsulated odontoblast-like cells (OD21), and the formation of endothelial monolayers by endothelial colony forming cells (ECFCs). GelMA hydrogels with higher polymer concentration (15% w/v) and stiffness enhanced OD21 cell viability, spreading and proliferation, as well as endothelial cell spreading and monolayer formation. We then fabricated pre-vascularized, full-length, dental pulp-like tissue constructs by dispensing OD21 cell-laden GelMA hydrogel prepolymer in root canals of extracted teeth and fabricating 500 µm channels throughout the root canals. ECFCs seeded into the microchannels successfully formed monolayers and underwent angiogenic sprouting within 7 days in culture. In summary, the proposed approach is a simple and effective strategy for engineering of pre-vascularized dental pulp constructs offering potentially beneficial translational outcomes.

## Introduction

Dental pulp is a highly vascularized, innervated, unmineralized connective tissue that occupies a chamber and long canal in the center of a semi-permeable tissue structure constituted of dentin tubules and mineralized matrix, spanning from the root apex through the crown. The formation of dentin, the tissue surrounding the pulp, is achieved by odontoblasts, which are specialized cells that are located in a pseudo-stratified layer at the periphery of the pulp chamber and root canal. Among other tissue components, such as fibroblasts, neurons, and resident stem cells, the pulpal tissue comprises a network of blood capillaries that traverse centrally through the pulp extending towards the tooth crown. Microcapillaries branching outwards from the core vessel form a capillary-rich plexus a few micrometers away from the odontoblast layer near the dentin^1–4^.

Root canal treatment is necessary in the event of deep caries or trauma when the homeostasis of the pulp tissue is lost. Current root canal treatment methods typically involve removal of infected or necrotic tissue and replacement with inert synthetic biomaterials, thus sacrificing the biological response of the tooth^5^. Regeneration of the pulp tissue to restore tooth function, a strategy that has been named regenerative endodontics, has been proposed as an alternative to conventional root canal therapy^6–8^. However, since the biological function of the pulp is primarily regulated via the existing vasculature, novel strategies that allow for controlled regeneration of vascularized pulp are critically necessary^9^.

**Figure 1:**
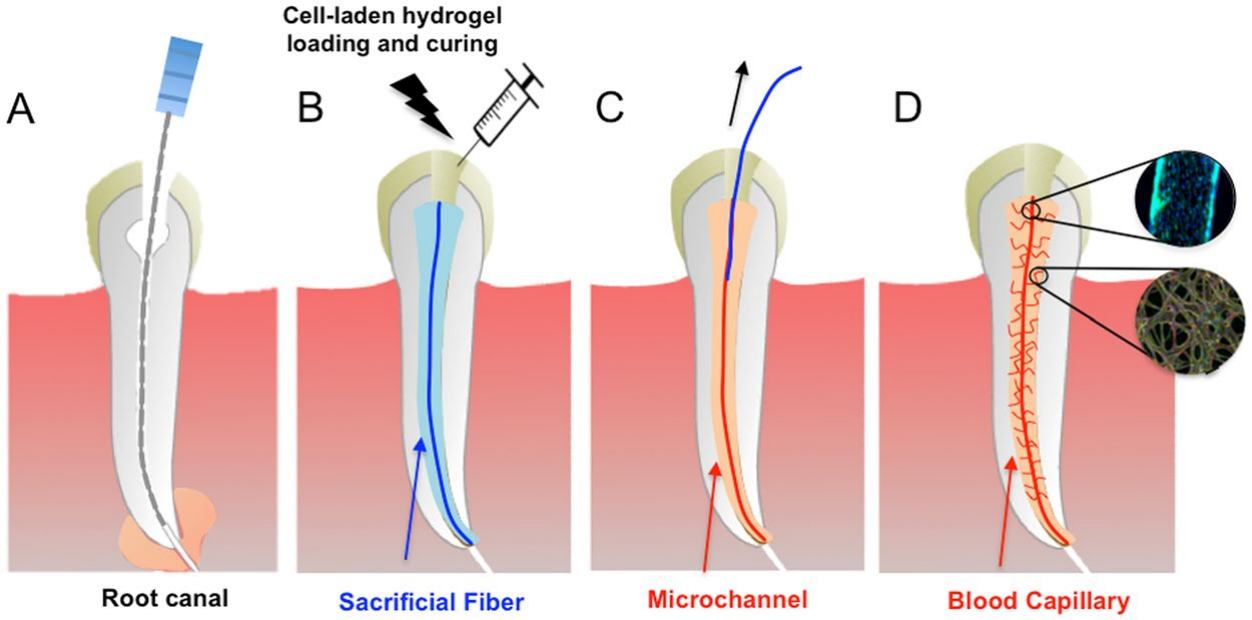
Schematic diagram illustrating the basic steps of the proposed strategy to engineer pre-vascularized full-length dental pulp-like tissue constructs. (**A**) The root canal is prepared following common endodontic procedure using endodontic files. (**B**) A pre-made sacrificial fiber is positioned in the root canal. A cell-laden hydrogel is loaded into the canal and photopolymerized. (**C**) After the hydrogel photopolymerization, the sacrificial fiber is removed, creating a hollow microchannel that traverses the entire length of the canal, from the apex through the pulp chamber. (**D**) Endothelial cells are seeded in the fabricated microchannel to engineer the core vascular capillary in the dental pulp, thus resulting in a pre-vascularized full-length dental pulp-like tissue construct. Details are provided in Supplementary Figure S1.

Vascularization is a process that relies on complexly orchestrated biological events, such as the morphogenesis of endothelial cells into new hollow capillaries (vasculogenesis), the recruitment of perivascular mural cells (pericytes), and the remodeling of the existing networks into a dense vascular plexus via angiogenic sprouting^10–12^. Several studies have shed light on the regeneration of vascularized pulp by culturing endothelial and/or stem cells on flat substrates^13, 14^, in three-dimensional (3D) scaffold matrices^13–21^, and in scaffold-less tissue constructs^22, 23^. In earlier noteworthy developments Sakai *et al*. reported that stem cells from exfoliated deciduous teeth (SHEDs) differentiated into endothelial cell networks in a poly-L-lactic acid scaffold in the presence of recombinant VEGF *in-vitro*, and in untreated scaffolds *in-vivo* ^20^. More recently, Dissanayaka *et al*. used a commercially available hydrogel (Puramatrix™) encapsulated with dental pulp stem cells (DPSCs) and human umbilical vein endothelial cells (HUVECs) to determine the role of DPSCs in the angiogenic process, and partially regenerate dental pulp in root canals implanted in the back of immunocompromised mice^14, 16^. However, these strategies require time intensive biological processes for a functional and interconnected vasculature to be formed. To date, a more simplified biofabrication strategy that is compatible with short-term vasculature formation and has greater clinical translational potential is lacking. This presents a significant hurdle towards the use of regenerative endodontics in clinical practice, especially for full-length root canals of mature teeth, where oxygen delivery is only achieved via the root apex. Therefore, to prevent hypoxic conditions in the tissue until neo-vasculogenesis occurs, we contend that an engineered vasculature that is present from the onset of the regenerative process represents an improved strategy for regeneration^24^ of vascularized dental pulp. To address these challenges, we present a novel strategy to fabricate pre-vascularized pulp-like hydrogel tissue constructs in full-length root canals *in-vitro* **(**Fig. 1A–D **)**.

We first evaluated the extracellular microenvironmental conditions that enhance the viability, spreading and proliferation of odontoblast-like cells (OD21) embedded in gelatin methacryloyl (GelMA) hydrogels having tuned physical properties by virtue of varying polymer concentrations. We then optimized the conditions promoting the formation of endothelial monolayers on GelMA hydrogel substrates with endothelial colony forming cells (ECFCs). Lastly, we provided proof-of-principle evidence for the engineering of endothelialized and pre-vascularized pulp-like tissue constructs in full-length root canals *in-vitro*. Although we report proof of principle experiments utilizing animal cell sources that are not compatible with direct clinical applications, and are only intended at initial screening of the developed protocols, we argue that the proposed approach may form the basis for a simple and effective strategy for the engineering of vascularized dental pulp with potentially beneficial translational outcomes.

## Results and Discussion

### Microstructure and physical properties of GelMA hydrogels

**Figure 2:**
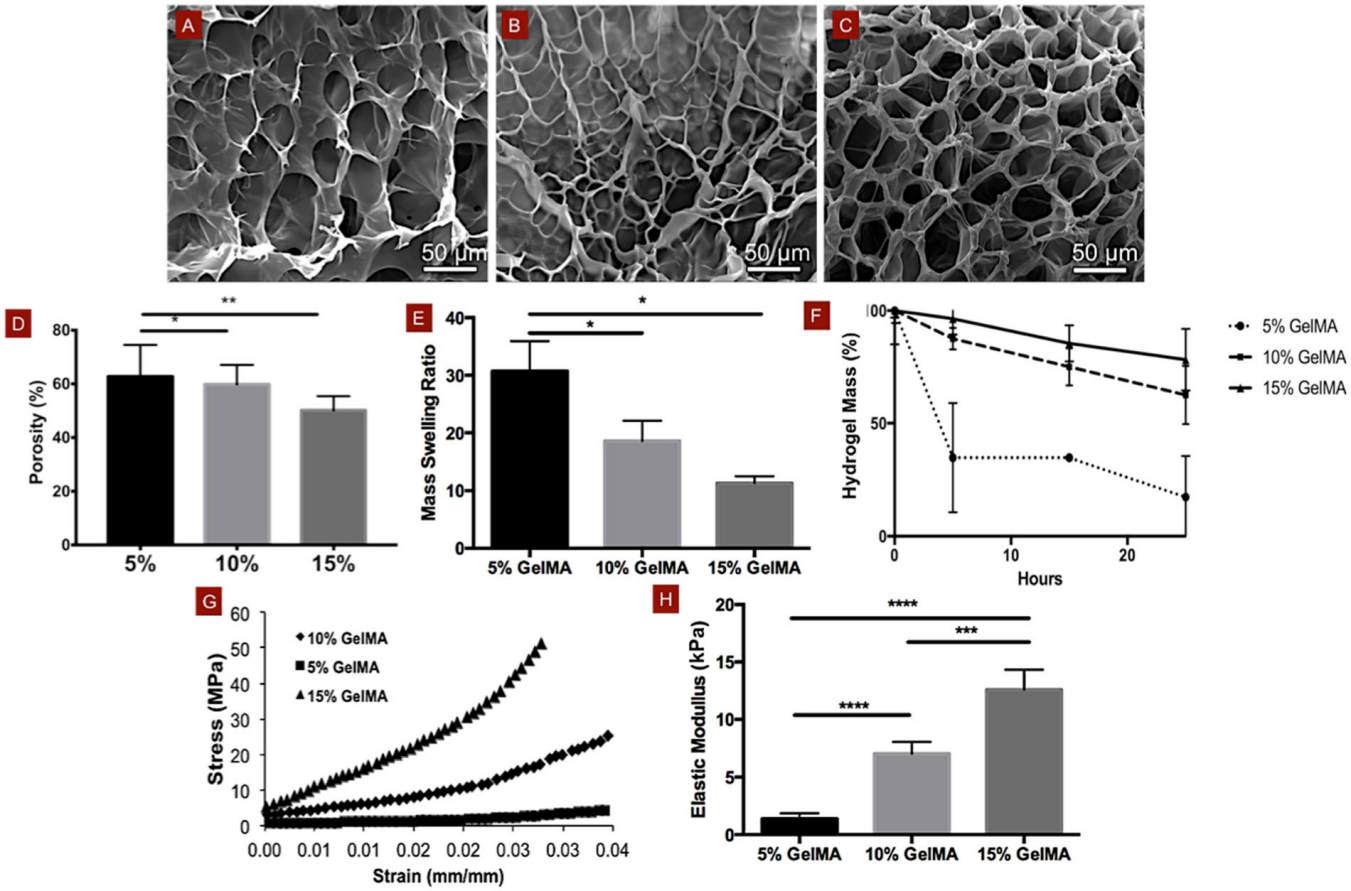
Physical and mechanical properties of GelMA hydrogels. SEM images of (**A**) 5%, (**B**) 10% and (**C**) 15% (w/v) GelMA hydrogels show a decrease in apparent pore sizes and (d) relative percentage of porosity with increasing hydrogel concentration, especially comparing 5% to 10 or 15% hydrogels. Mass swelling ratios and (**E**) degradation profiles of 5, 10 and 15% GelMA hydrogels in DPBS, and in 2.5 U ml^–1^ collagenase, respectively showing a marked decrease in swelling properties and degradability with increasing polymer volume fraction. (**F**) Stress-strain curves and (**G**) elastic modulus for 5, 10 and 15% GelMA hydrogels in unconfined compression, respectively demonstrate enhanced mechanical properties in more densely crosslinked hydrogels (15%). For degradation data, p < 0.0001 between 5% and 10%, and between 5% and 15% for time points 15 and 25 h. Statistical significance is represented by *for p < 0.05, **for p < 0.01, ***for p < 0.001 and ****for p < 0.0001.

GelMA hydrogels have been extensively utilized for a variety of tissue engineering applications (see Yue *et al*.^25^ for a recent review), however to the best of our knowledge, these scaffold materials remain little explored regarding their application for dental pulp regeneration^26^. The physical properties of hydrogels such as porosity, degradability, swelling and mechanical properties are influenced by the nature and extent of crosslinking of the polymer during gelation, and are known to affect cell behavior and function during tissue formation^27^. SEM analysis (Fig. 2A–C) of cross-sectioned GelMA hydrogels of 5, 10 and 15% (w/v) concentrations showed a honeycomb-like structure in all three groups. Both 10% and 15% hydrogel groups appeared to have smaller pore sizes (Fig. 2B,C) than 5% (Fig. 2A) GelMA, thus indicating the formation of denser crosslinked networks in hydrogels with higher concentrations. The relative percent porosity of the hydrogels (Fig. 2D), which is indicative of total area of pores relative to that of polymer in the material, showed a significant decrease in total pore area from 5% hydrogel compared to both 10, and 15% hydrogels, respectively. The rate of diffusion of a rhodamine dye solution through each of these hydrogels corresponded with their apparent pore sizes (Supplementary Figure S2), where 5% GelMA hydrogels were visibly more permeable. Also, the increased polymer volume fraction correlated with increased thickness of the pore walls. However, the pore sizes of 10% and 15% GelMA hydrogels were not noticeably different from one another. Recent work has also analyzed the pore structure of GelMA hydrogels using the same synthesis protocols utilized here and reported the same trends we found^25, 28^. We then sought to determine the effect of hydrogel concentration on the swelling, degradation and mechanical properties of the hydrogel scaffolds to enable a more thorough analysis of the behavior of the ECFC and OD21 cells on these scaffolds.

Transport of biological molecules in microporous hydrogels occurs almost solely through diffusion of solutes, thus the capacity of a hydrogel to swell is indicative of the diffusivity of fluids through the scaffold^29^. 5% GelMA hydrogels had significantly higher mass swelling ratios (Fig. 2E) which were nearly two- and three-fold higher than those of 10% and 15% GelMA hydrogels, respectively, which is consistent with the pore size structure seen in our SEM images. 5% GelMA hydrogels were also more susceptible to degradation by collagenase (Fig. 2F) and underwent 83% degradation within a 24-hour period, while 10% and 15% GelMA hydrogels were degraded to an extent of only 38% and 22% respectively. Although hydrogel scaffold remodeling in the dental pulp is likely driven by pulp-specific collagenases, such as MMPs 1, 8 and 13^30, 31^, hydrogel degradation with unspecific collagenases has also been shown to interfere with important mechanisms of cell differentiation. Cell-mediated degradation of hydrogels has been shown to influence stem cell differentiation through cell spreading and cellular traction, with higher degradability prompting osteogenic phenotypes in human Mesenchymal Stem Cells (hMSCs)^32^. Interestingly, our studies showed that OD21 cells encapsulated in 10% and 15% GelMA hydrogels were more spread than those in 5% GelMA hydrogels, therefore suggesting a closer correlation between cell spreading and hydrogel elasticity than cell spreading and hydrogel degradation.

Unconfined compression of the 5, 10 and 15% hydrogels revealed a steeper stress-strain curve (Fig. 2G) and a correspondingly higher elastic modulus (Fig. 2H) for the higher concentration gels (12 kPa) in comparison to the 10% (7 kPa) and 5% (1.5 kPa) gels. The higher polymer concentration of GelMA hydrogels appeared to have a more profound effect on the elasticity than degradation, porosity or swelling, which were comparable between 10 and 15% groups. Therefore, the following discussion will focus primarily on the relationship between matrix elasticity and OD21 and ECFC behavior.

### OD21 cell viability, spreading and proliferation in GelMA hydrogels

**Figure 3:**
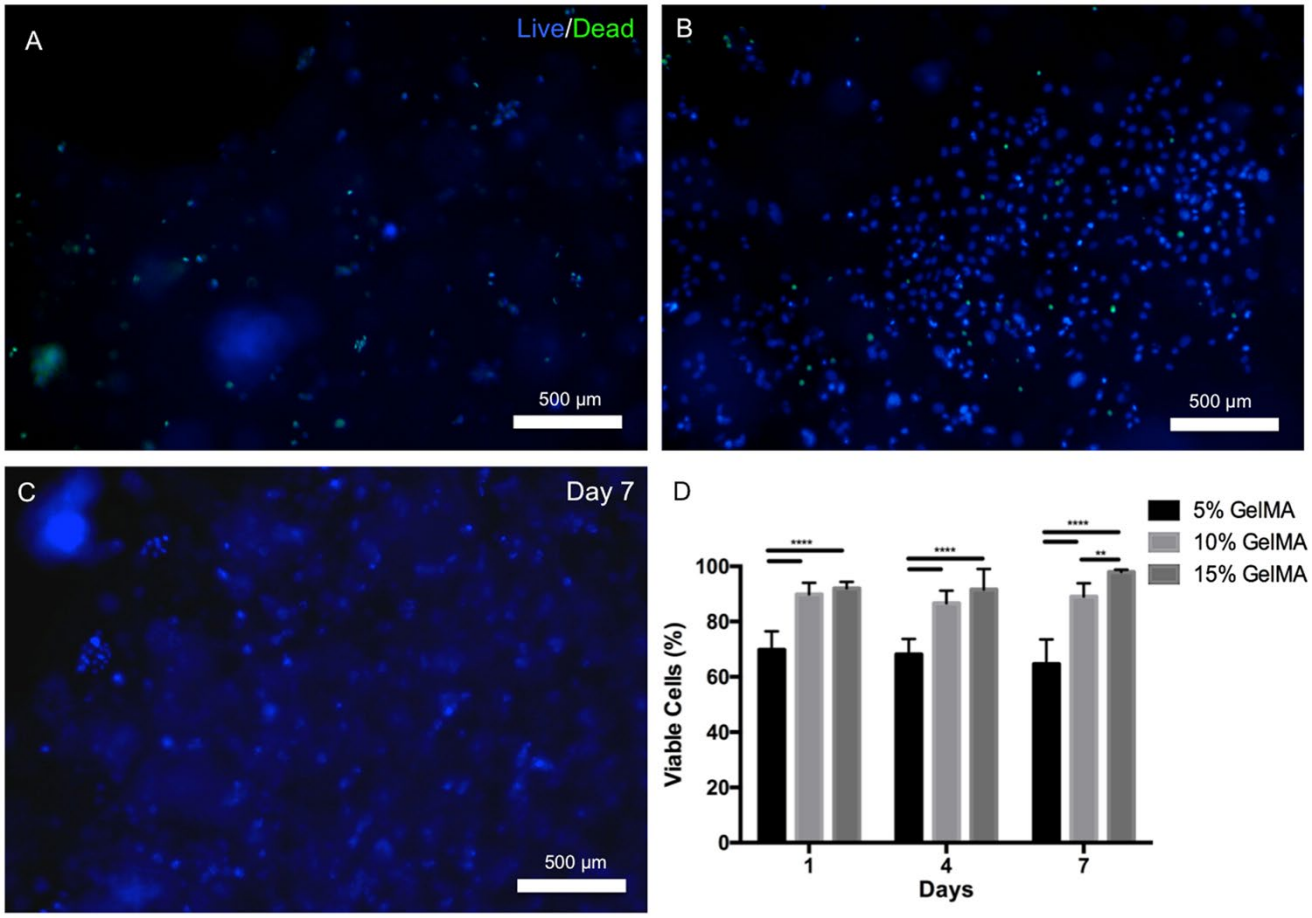
Viability of OD21 cells in GelMA hydrogel scaffolds. Representative images of OD21 cells encapsulated in (**A**) 5%, (**B**) 10% and (**C**) 15% GelMA hydrogels stained for live (blue) and dead (green) cells on day 7. (**D**) Percentage of live cells in the hydrogels after 1, 4 and 7 days showed consistently enhanced OD21 cell survival in 10 and 15% GelMA hydrogels compared to the softer 5% GelMA hydrogels at all time points, with a statistically significant increase in cell viability in 15% GelMA hydrogels over 10% GelMA hydrogels by day 7. Statistical significance is represented by **for p < 0.01 and ****for p < 0.0001.

OD21 cells encapsulated in 5, 10 and 15% GelMA hydrogels showed higher survival rates in the stiffer gels even at early time points and the effect was sustained through the 7 day period of the study (Fig. 3A–D). At later time points, cells displayed a statistically significant preference for 15% over the 10% GelMA hydrogels. Our ^1^H NMR data showed a methacrylation efficiency of 87.8 ± 3.3% (Supplementary Figure S3), which is consistent with previous reports using the synthesis protocols we utilized here^28, 33^. However, it is difficult to conclude if the lower viability reported for OD21 cells encapsulated in the 5% formulation is really a response of the cells to the stiffness of the hydrogels alone, as the presence of unreacted photoinitiator relative to the available methacrylates in the 5% GelMA formulation may also have contributed to the lower viability of OD21 cells in this group. Our attempts to measure the final degree of conversion of methacrylates in our photocrosslinked GelMA hydrogels to confirm such hypothesis were unsuccessful (data not shown), and this represents a limitation of this study.

**Figure 4:**
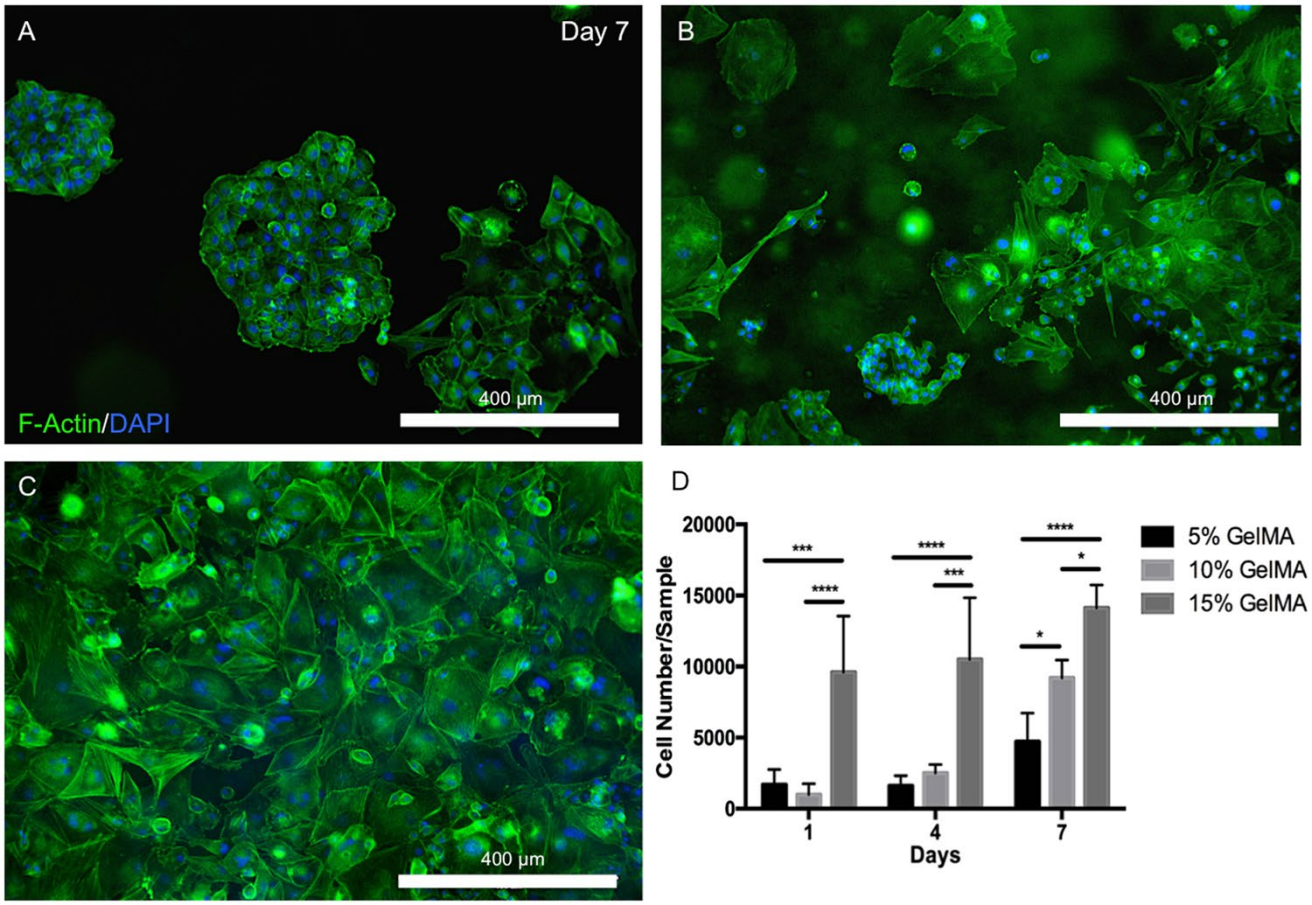
Spreading and proliferation of OD21 cells in 3D GelMA hydrogels. Representative images of OD21 cells encapsulated in (**A**) 5%, (**B**) 10% and (**C**) 15% GelMA hydrogels stained for actin (green) and DAPI (blue) shows increased cell spreading in stiffer over softer hydrogels. (**D**) Quantification of the number of cells per gel after 1, 4 and 7 days in culture indicates a significantly higher rate of proliferation of OD21 cells in scaffolds of higher polymer concentration. Statistical significance is represented by *for p < 0.05, ***for p < 0.001 and ****for p < 0.0001.

The cells in the stiffer (15%) gels were more spread which translated to a three times higher rate of proliferation over a 7 day period than that in softer gels (5%) (Fig. 4A–D). We also observed that the cells closest to the substrate support in the cell-laden hydrogels were more spread, which further suggests the propensity of this cell line for higher spreading near stiffer matrices or substrates. This is in keeping with what is known about cell responses to matrix elasticity, since the OD21 cells appeared to thrive in stiffer matrices like the lightly calcified pre-dentin at the periphery of the dental pulp, the site of these cells in native tissues^3, 4^.

### Effect of GelMA hydrogel elasticity on monolayer formation by ECFCs

**Figure 5:**
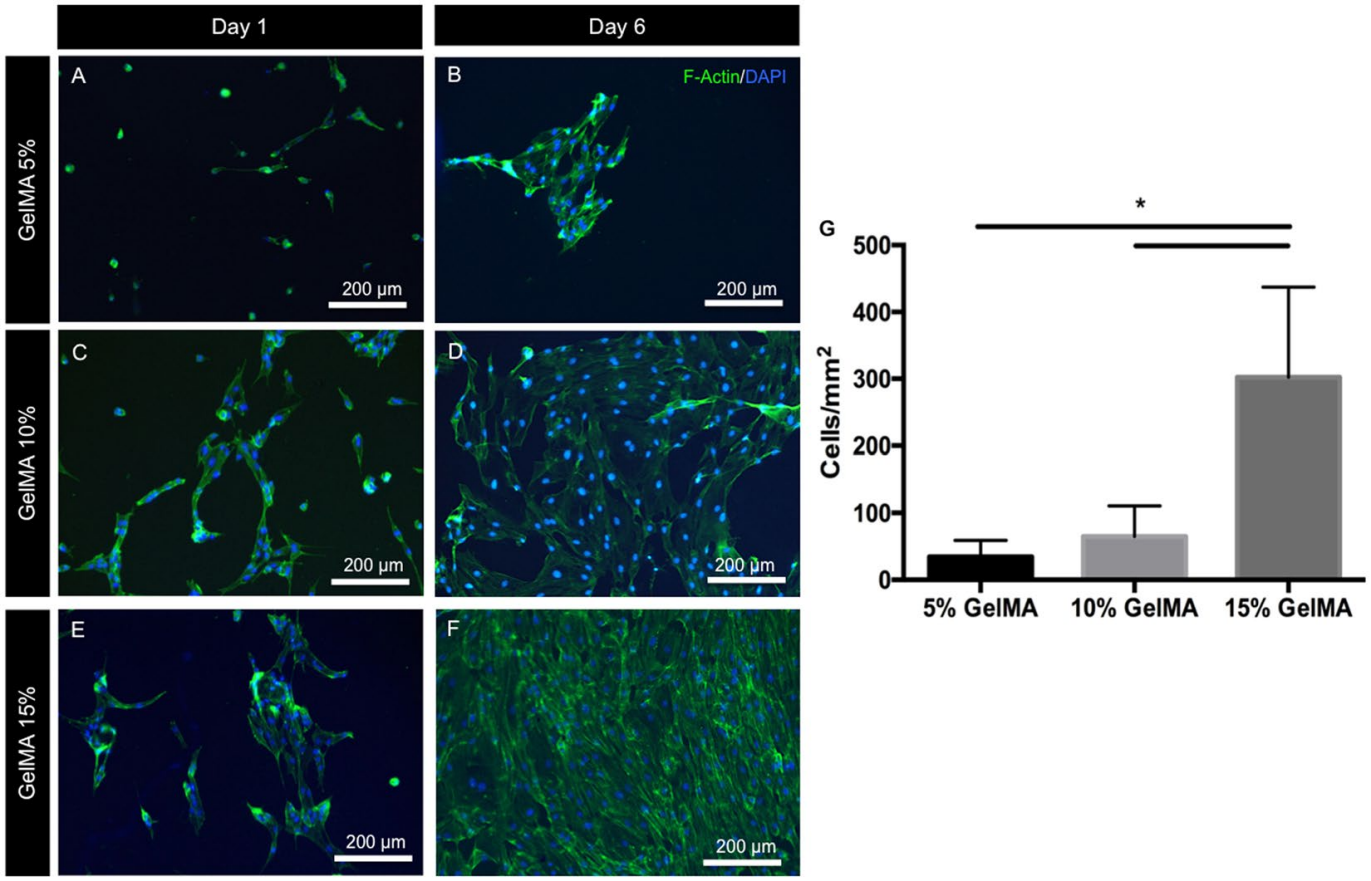
Cell spreading and monolayer formation by ECFCs on 2D GelMA hydrogels. Representative images of ECFCs on (**A**,**B**) 5%, (**C**,**D**) 10% and (**E**,**F**) 15% GelMA hydrogels after 1 and 6 days in culture, stained for actin (green) and DAPI (blue) showed increased cell spreading and cell density on stiffer substrates at both early and late time points. (**G**) Quantification of the number of cells per unit surface area on day 6, representative of cell coverage on the hydrogels and the propensity for endothelial monolayer formation suggests that stiffer GelMA hydrogel substrates support ECFC proliferation and monolayer formation. Statistical significance is represented by *for p < 0.05.

Vascular cell behavior is modulated by the mechanical properties exerted by the arterial membrane^34^. The endothelial cells forming the blood vessels are in contact with and influenced by the tensile forces exerted by the basal and outer membranes of the arterial walls comprising the extracellular matrix and elastin. In addition, these cells are constantly subjected to shear stresses due to fluid flow within the vessels. The combination of tensions and shear stresses exerted on the endothelial cells influence their proliferation and monolayer formation through biochemical cues^35^. ECFCs cultured on flat 2D substrates (Fig. 5A–F) and in microchannels (Supplementary Figure S4) in 5, 10 and 15% GelMA hydrogels showed increased proliferation and a greater tendency to form monolayers in gels of higher stiffness. Previous studies on the effect of substrate stiffness on endothelial cells have observed that soft substrates were more conducive to microvascular network formation^36^, while stiffer matrices supported endothelial cell proliferation^37^, individual cell spreading and hence monolayer formation^38^.

In summary, our results (Figs 3–5) suggest that relatively stiffer (12 kPa) GelMA hydrogels enhance both OD21 cell behavior as well as endothelial monolayer formation by ECFCs. Therefore, we chose to employ 15% GelMA hydrogels in our proposed strategy to engineer pre-vascularized pulp-like tissue constructs.

### Fabrication of pre-vascularized hydrogel scaffolds for pulp regeneration *in-vitro*

In order to test the above observations in hydrogel scaffolds with physiological dimensions and constraints, we employed a full-length root canal model wherein we fabricated a microchannel in cell-laden GelMA hydrogel tissue constructs. The rationale behind the proposed strategy was to ensure that a functional vascular-like conduit could be formed through the engineered pulp scaffolds from the onset of the regenerative process. This, in turn, should ensure that oxygen/nutrient diffusion and waste removal is optimized through the length of the scaffolds during the remodeling process. Moreover, the fabricated microchannel should provide a path for the migration of host cells to home into the scaffold structure from the root apex. It is noteworthy that complete and effective regeneration of the dental pulp would require not only engineering of the pulp vasculature, but rather all of the cell and tissue components that make up the tissue structure, including nerves, fibroblasts, stem cells, immune cells and others. Nevertheless, here we adopted a reductionist approach that would allow us to study primarily the vascular and odontoblast component of an engineered tissue, with especial emphasis on the feasibility of the biofabrication method that we designed.

**Figure 6:**
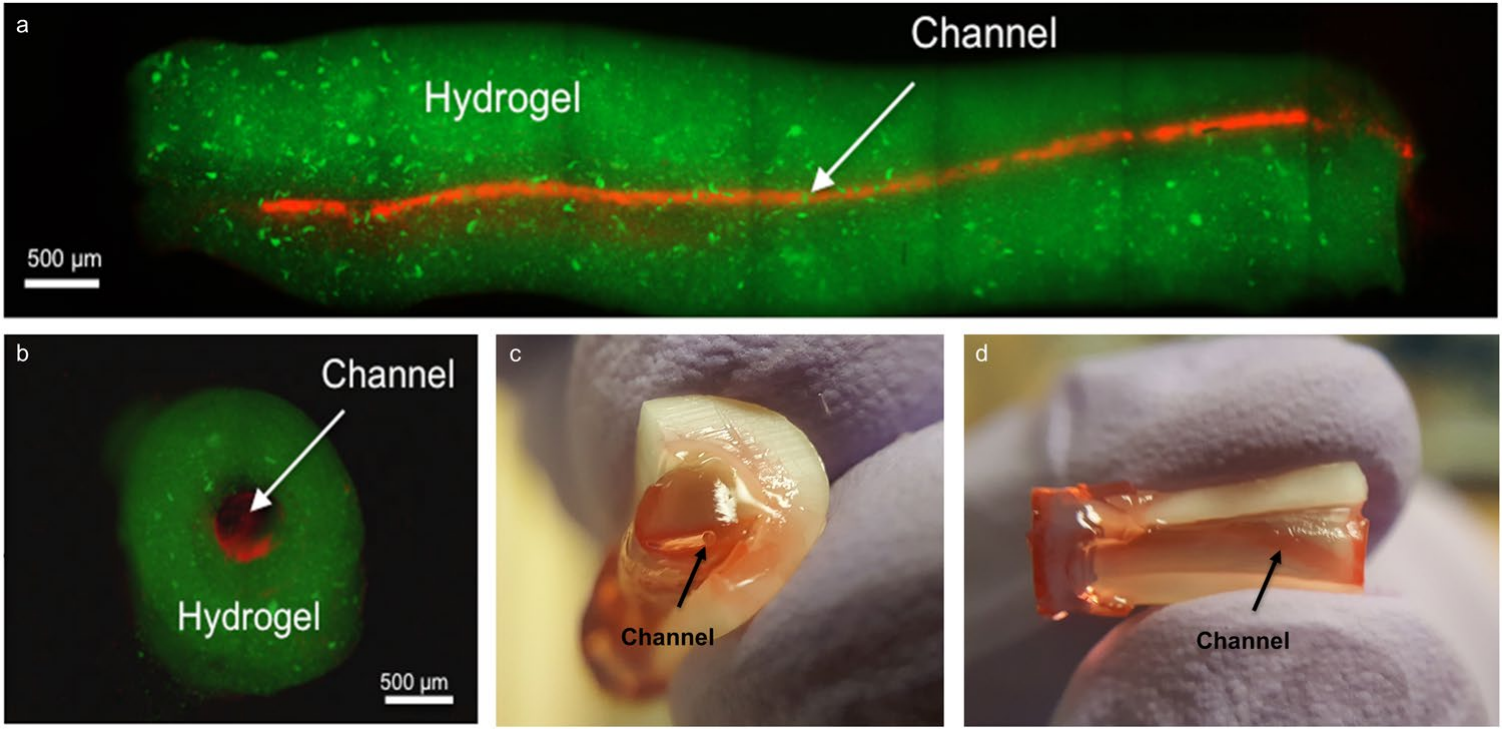
Representative images of pre-vascularized pulp-like tissue construct. (**A**) Longitudinal and (**B**) cross-sectional views of GelMA hydrogels loaded with green fluorescent microparticles showing the fabricated microchannel after being perfused with a red fluorescent microparticle solution. The channels cross the entire length of the root. (**C**,**D**) Photographs of GelMA hydrogels from longitudinal and occlusal perspectives inside a full-length root fragment. Root fragments were stabilized prior to hydrogel loading and microchannel fabrication, and were separated to retrieve the constructs and illustrate the position of the hydrogel inside the tooth. Microchannels were perfused with red food dye.

To better approximate the clinical situation, we used extracted, endodontically-prepared, single-rooted human pre-molars and positioned an agarose fiber across the entire length of root canal, traversing from the apex through to the cervix. We then loaded a cell-laden 15% GelMA hydrogel precursor up to 3 mm of the root length and exposed the tooth to UV light for 30 s. We repeated the hydrogel precursor loading and curing process 3 times, to ensure thorough photo-polymerization of the material (Supplementary Figure S5) along the entire root canal, while ensuring that the cells were exposed to the same intensity of UV light that we determined to be non-cytotoxic in our cell-viability experiments. It may be worth noting that the coronal loading of both the hydrogel and the ECFCs into the root canals represent advantages for future clinical translation. Figure 6 shows representative fluorescent microscope images and photographs of a fluorescently labeled (green) (Fig. 6A,B) hydrogel tissue construct that was retrieved from an endodontically prepared tooth, after perfusion of the fabricated microchannel with a red-fluorescent particle solution (Fig. 6A,B) or a red dye (Fig. 6C,D). The longitudinal and transversal position of the microchannel along the tooth are visible and notably not symmetrically positioned within the center of the canal, which represents a limitation of the proposed method that we are currently optimizing. Supplementary Figure S6 depicts photographs of an intact cell-laden hydrogel tissue construct containing a microchannel retrieved from a similarly endodontically prepared tooth after 7 days (Supplementary Figure S6a,b) in culture and a phase contrast microscope image (Supplementary Figure S6d) where densely cellularized structures are visible both within and outside the microchannel. Figure 1 shows a schematic diagram illustrating basic steps of the proposed strategy to engineer pre-vascularized full-length dental pulp-like tissue constructs.

**Figure 7:**
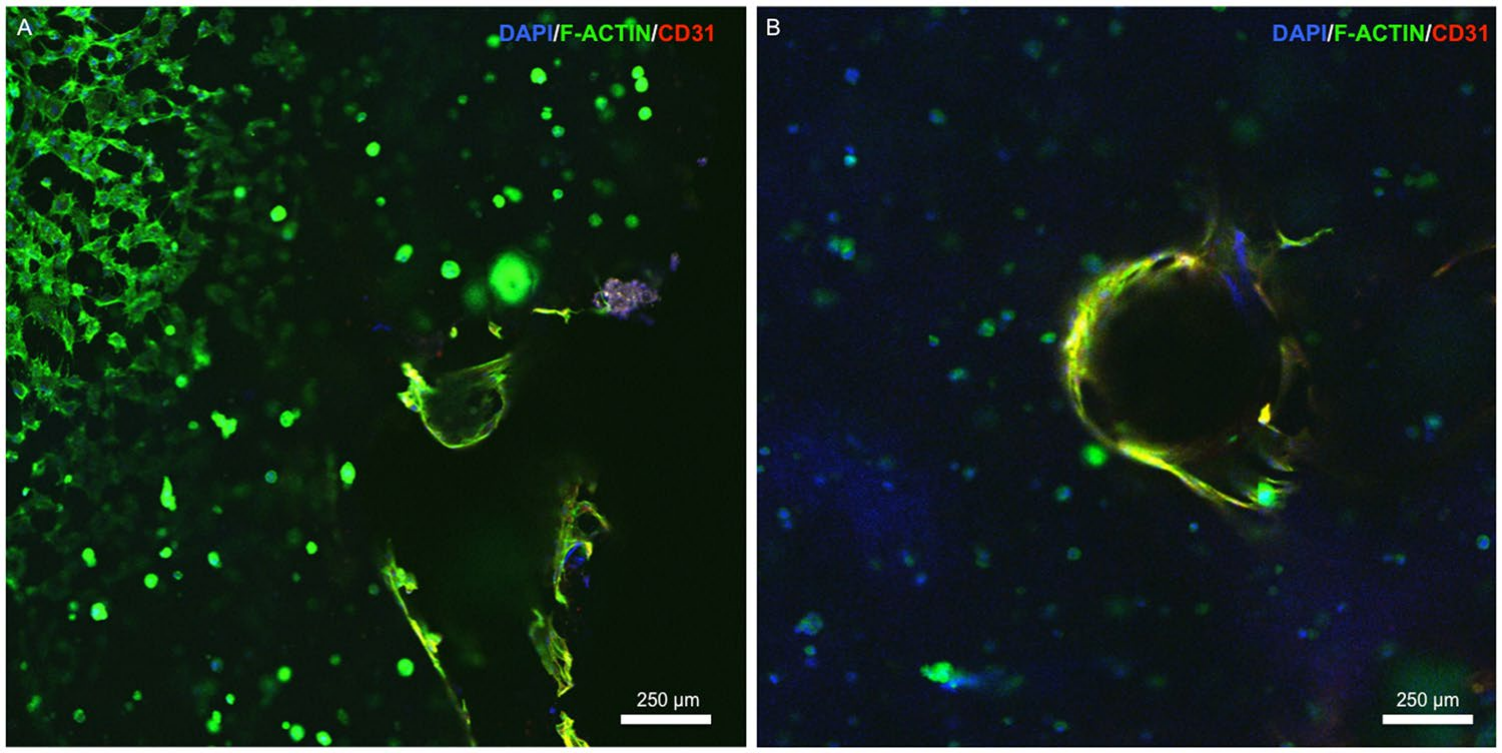
Confocal images of OD21 and ECFCs cultured in GelMA hydrogels in the full-length dental pulp-like tissue constructs. (**A**) OD21 cells had visibly higher spreading near the dentin walls (upper left corner of (**A**)) than in areas around the fabricated microchannels (**B**). Cells were stained for actin (green), DAPI (blue) and CD31 (red) on day 7. Z-stack movie for 3D rendering of this image is available as Supplementary Video 1.

**Figure 8:**
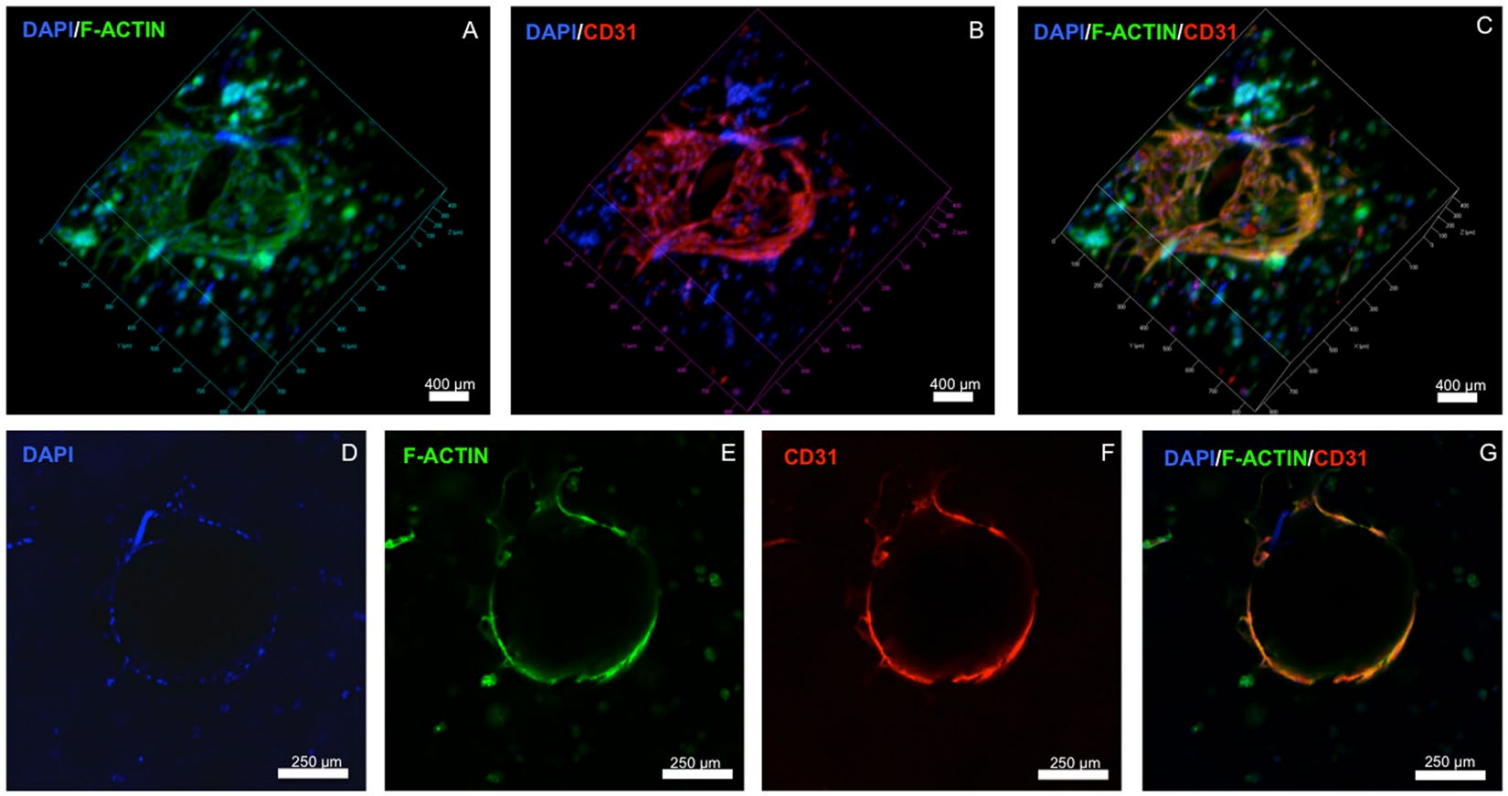
Confocal images of endothelialized microchannels in an OD21-laden GelMA hydrogel cultured in a full-length dental pulp-like tissue constructs. (**A**–**C**) 3D rendering and (**D**–**G**) cross-sectional slices of confocal images showing ECFC monolayer formation and angiogenic sprouts in the engineered microchannel in the dental pulp-like tissue constructs on day 7. Cells were stained for actin (green), DAPI (blue) and CD31 (red). Z-stack movie for 3D rendering of this image is available as Supplementary Video 2.

Interestingly, similar to the behavior we observed in our earlier experiments, confocal microscopy images of scaffolds retrieved from the prepared teeth showed that OD21 cells encapsulated within the hydrogels tended to have higher spreading in close proximity to the dentin walls (Fig. 7A) than around the microchannels (Fig. 7B), despite the visible presence of spread ECFCs in the form of endothelial sprouts in those areas. This further suggests the preference of OD21 cells for substrates of higher stiffness, as noted earlier. Figure 8(A–C) show a confocal image of the endothelial monolayer along the circumference of the microchannel in a section of the channel. CD31 marker is highly expressed in these cells, indicative of tight cell-cell junctions^39^. Cross-sectional views of these channels (Fig. 8D–G) also show endothelial cell sprouting in certain locations indicative of activation of angiogenic events through cell-cell junctions after monolayer formation as well as the dentin matrix components, which have been shown to stimulate angiogenic activity at relatively low concentrations^18, 20, 40^.

It is also noteworthy that although the current strategy is demonstrated here using GelMA hydrogels, we have previously shown that the proposed method can be used with a variety of different hydrogels, such as PEGDA, PEGDMA, SPELA^12^, as well as non-photo-crosslinkable materials, such as collagen (Supplementary Video 3). Moreover, the fact that the proposed photo-crosslinkable material can also be photocured using a conventional dental curing light^41^ makes this strategy more readily translatable from a clinical standpoint. Despite these interesting observations, it must be pointed out that, at this point, the proposed method does not yet reproduce the highly intricate density and morphology of the pulp micro-vasculature, or the presence of other important tissue components, such as nerves, fibroblasts, undifferentiated stem cells and others. Moreover, an important limitation of this study that must be highlighted is that we utilized cells isolated from different animal species, which may not be consistent with the behavior of human dental pulp cells, and also could lead to undesirable inter-specifies phenotypic interactions that affect the immediate translation of this method. Despite these obvious limitations, we are convinced that the engineered microchannels could enhance the formation of cell-based microvessels in a hydrogel loaded with co-cultures of endothelial cells and dentin progenitor cells of human source. This forms the basis for future studies in our laboratory, as well as a more thorough *in-vivo* analysis of the effectiveness of the proposed approach.

## Conclusion

In this report, we demonstrate a novel strategy to engineer pre-vascularized pulp-like tissue constructs using tunable cell-laden GelMA hydrogels. Our results suggest that OD21 cells encapsulated in hydrogels of higher stiffness have higher spreading, proliferation and viability. Similarly, ECFCs showed greater spreading and tendency to form endothelial monolayers when seeded on stiffer gels. Furthermore, we provide proof-of-principle evidence for the fabrication of pre-vascularized cell-laden pulp-like tissue constructs in a full-length length root canal *in-vitro*. In our root canal model, OD21 cells showed greater spreading and proliferation near the dentin walls, while ECFCs formed endothelial monolayers with active angiogenic sprouts in fabricated microchannels after 7 days in culture. In summary, this approach represents a simple, yet effective, strategy to engineer pre-vascularized dental pulp-like tissue constructs with highly translational outcomes.

## Materials and Methods

### Gelatin methacryloyl (GelMA) synthesis

GelMA was synthesized following previously published protocols^33^. Briefly, 10% (w/v) type A gelatin from porcine skin (Sigma) was dissolved in Dulbecco’s phosphate buffered saline (DPBS, Sigma). The solution was stirred and heated to 50 °C and 8% (v/v) methacrylic anhydride (Sigma) was added to the solution in a dropwise manner. The reaction was allowed to proceed for 2 hours at 50 °C before being stopped using a 5x dilution of 40 °C DPBS. The resulting solutions were dialyzed against distilled water using 12–14 kDa dialysis tubing at 45 ± 5 °C for five days with two water changes per day. The solution was then stored at −80 °C overnight and lyophilized for 5 days prior to use. A methacrylation efficiency of 87.8 ± 3.3% (Supplementary Figure S3) was confirmed by ^1^H NMR following published protocols^33^. We refer the reader to a recent review and detailed protocol describing the chemistry, synthesis, characterization and applications of GelMA hydrogels^25, 28^.

### Hydrogel preparation

GelMA macromer at concentrations of 5, 10 and 15% (w/v) was dissolved in DPBS with 0.1% (w/v) 2-hydroxy-4′-(2-hydroxyethoxy)-2-methylpropiophenone (Tokyo Chemical Industries) photoinitiator. GelMA hydrogel discs measuring 7 mm in diameter, were fabricated by dispensing the hydrogel precursors in PDMS molds and exposing samples to UV light (320–390 nm) (EXFO Acticure 4000) with a power of 850 mW for 30 seconds at a distance of 8.5 cm.

### Physical and mechanical characterization

Hydrogel pore structure and morphology was analyzed via scanning electron microscopy. To that end, 5, 10 and 15% (w/v) GelMA hydrogel disks (n = 3) were prepared as described above, cross-sectioned, flash frozen in liquid nitrogen, fixed using a solution containing 2% Paraformaldehyde, 2.5% Glutaraldehyde for two hours, and lyophilized overnight. Samples were then coated with gold/palladium and imaged using a FEI Quanta 200 SEM at 20.0 kV. Quantification of relative percent of porosity was performed using ImageJ, after converting SEM micrographs into binary images and determining the pore area relative to the total area in the image; a minimum of 11 images were analyzed per group (n = 3). For swelling analyses, 5, 10 and 15% (w/v) GelMA hydrogel discs (n = 6) were stored for 24 hours at room temperature in DPBS, removed from the solution, blot dried, and the swollen weight recorded. The dry weights of the samples were collected after sample lyophilization and the mass swelling ratio was calculated as the ratio of the wet mass to the dry mass of the polymer. Hydrogel degradation was determined by incubating GelMA hydrogel disks (n = 6) for 5, 12 and 24 hours at 37 °C in a 2.5 U ml^−1^ collagenase solution (MP Biomedical). After incubation, the non-degraded hydrogel fragments were retrieved and the solution removed. Samples were then rinsed with DPBS before removal of excess liquid and lyophilization overnight. Degradation percentage was determined by calculating the weight ratio of degraded versus intact hydrogel samples at each time point. Lastly, hydrogel elastic modulus was tested in unconfined compression at a loading rate of 1 mm min^−1^ on a universal mechanical testing machine (Instron 5542). Prior to testing 5, 10 and 15% (w/v) GelMA hydrogel disks (n = 6) were stored in DPBS for 24 h. Samples were then blot dried and the elastic modulus of the hydrogel was determined as the slope of the linear region corresponding to 0%–10% strain.

### Cell culture

To determine the behavior of odontoblast-like cells in the engineered hydrogels, we utilized an odontoblast-like cell line (OD21)^42^. OD21 cells were cultured in DMEM containing 10% (v/v) fetal bovine serum (FBS) and 1% (v/v) penicillin-streptomycin. To study endothelial monolayer formation, we utilized primary baboon endothelial colony forming cells (ECFC) isolated from peripheral blood using previously established protocols^43^. ECFCs from passage 5–6 were cultured in endothelial cell growth medium (EGM-2 MV, Lonza), containing 5% (v/v) fetal bovine serum (FBS) and 1% (v/v) penicillin-streptomycin. All cells were maintained in a humidified, 37 °C, 5% CO_2_ incubator, and the media changed every two days with two cells passages per week for OD21 and once per week for ECFC.

### OD21 cell encapsulation, viability, proliferation and spreading

Cell-laden hydrogel constructs were fabricated by dispensing 5 μl of a cell-laden GelMA hydrogel precursor (5 × 10^6^ cells ml^−1^) on TMSPMA coated glass slides. The hydrogel precursor was then compressed to 100 µm thick disks and photo-crosslinked as described above. The viability of OD21 in 5, 10 and 15% (w/v) GelMA hydrogels was observed using a membrane permeability based live/dead assay kit (Molecular Probes). The live and dead cells were counted using ImageJ software using at least 3 locations of triplicate samples after 1, 4, and 7 days. The percentage of viable cells was then calculated based on the number of live cells divided by the total cell number. Cell proliferation was determined using an ActinGreen/NucBlue assay kit (Molecular probes), for which the constructs were first fixed in 4% (v/v) paraformaldehyde (Electron Sciences) for 30 min, permeabilized in 0.1% (w/v) Triton X-100 solution for 20 min and blocked in 1% (w/v) bovine serum albumin (BSA) for 1 h. Samples were then incubated in the ActinGreen staining solution for 45 min at room temperature, and in a NucBlue staining solution for 10 min at 37 °C to stain the cell nuclei. Samples were imaged using either an inverted fluorescence microscope (FL Auto, Evos) or a laser scanning confocal microscope (ZEISS Airyscan LSM 880). The number of cells in each sample was computed using ImageJ software in three samples per group after 1, 4 and 7 days.

### ECFCs, spreading and monolayer formation

To study the formation of endothelial monolayers on GelMA hydrogels of different physical and mechanical properties, ECFCs (1 × 10^4^ cells ml^−1^) were seeded on top of pre-molded hydrogels of 5, 10 and 15% (w/v) concentrations. Endothelial cell coverage was calculated by counting the number of cells per mm^2^ on ImageJ. Cell spreading was qualitatively evaluated using an ActinGreen/NucBlue assay kit as described above.

### Fabrication of pre-vascularized dental pulp-like tissue constructs

Human pre-molars (n = 3) that were extracted for orthodontic reasons following protocols approved by the institutional review board (IRB) on research ethics were sectioned into 9 mm long root fragments having approximately 1.5 mm apical foramen diameter. Root fragments were UV sterilized (800 ± 10 mW, 10 min) and immersed in a 1% (v/v) penicillin-streptomycin solution for 24 h. Root canals in the tooth fragments were then prepared into a conical shape and sectioned longitudinally to allow for easier retrieval of the hydrogel samples after tissue culture (Fig. 1A). After sectioning, the two root halves were re-attached and secured by wrapping them with using laboratory film (Parafilm M). The root fragments were irrigated with 5 ml of 17% (w/v) EDTA^44^ to expose the bioactive molecules sequestered within the dentin^40^. To fabricate the microchannels, 500 µm diameter 6% (w/v) sacrificial agarose fibers were prepared using a glass capillary fitted with a metallic piston inside, using a 3D printing-inspired method we have developed recently^12^. The pre-solidified sacrificial fibers were manually positioned inside of the tooth, and the laboratory film was used to support the fibers in approximate center of the tooth (Fig. 1B). Based on the observation that 15% GelMA hydrogels lead to the optimal spreading of OD-21 and monolayer formation of ECFC cells, the 15% GelMA hydrogel precursor was chosen for proof-of-principle fabrication of the pre-vascularized dental pulp model. OD-21 cell-laden hydrogel precursor was loaded into the pulp chamber/root canal to fully surround the agarose fiber (Fig. 1C). The tooth fragment was filled 3 mm at a time to ensure homogenous photopolymerization of the hydrogel precursor. The detailed fabrication process is described in Supplementary Figure S1. After photopolymerization of the GelMA hydrogel, the agarose fiber was aspirated from the hydrogel using a light vacuum and a glass pipette, as previously described^12^. ECFCs were then seeded into the fabricated microchannels, while the construct was oriented horizontally in 4 separate seeding events (1 × 10^6^ cells ml^−1^ per seeding) with 1 hour between seedings to allow for cell attachment. Tissue constructs were then cultured in a combined DMEM and EGM-2 MV medium at a proportion of 1:1. After 7 days of culture, hydrogel samples were retrieved from the tooth, stained with ActinGreen/NucBlue, and immunostained for CD31. Briefly, the samples were fixed in 4% (v/v) paraformaldehyde for 20 min and permeabilized in 0.1% (v/v) Triton X-100 for 30 minutes. Samples were then blocked with 1.5% (w/v) bovine serum albumin for 1 hour at room temperature and exposed to a 1/50 dilution of mouse monoclonal antibodies against CD31 (Dako). The gels were washed in DPBS three times before the addition of a 1/250 dilution of the secondary antibody, conjugated goat anti-mouse (Thermo Scientific) and allowed to incubate at room temperature for 2 hours followed by copious rinsing with DPBS prior to imaging using a confocal laser scanning microscope.

### Statistics

Statistical analysis was performed using GraphPad Prism 6 for sections 2.1–2.3. The values represent averages ± standard deviations. One-way/two-way ANOVA was used to analyze the differences between GelMA concentrations and culture time followed by Tukey post-hoc tests (α = 0.05).

## Electronic supplementary material

Video 1

Video 2

Video 3

Supplementary Info
