# Supplementary material for: A Novel Strategy to Engineer Pre-Vascularized Full-Length Dental Pulp-like Tissue Constructs
Source: Sci Rep. 2017 Jun 12;7:3323. doi: 10.1038/s41598-017-02532-3 (PMC5468292; doi:10.1038/s41598-017-02532-3)
Supplement: Supplementary file 4 — Supplementary Info [file 41598_2017_2532_MOESM4_ESM.doc]

**A Novel Strategy to Engineer Pre-Vascularized Full-Length Dental Pulp-like Tissue Constructs**

Avathamsa Athirasala^1#^, Fernanda Lins^1#^, Anthony Tahayeri^1^, Monica Hinds^2^,Anthony J. Smith^3^, Christine Sedgley^4^, Jack Ferracane^1^, Luiz E. Bertassoni^1,2,5,6*^

^1^Division of Biomaterials and Biomechanics, Department of Restorative Dentistry,
School of Dentistry, Oregon Health and Science University, Portland, OR, USA
^2^Department of Biomedical Engineering, School of Medicine, Oregon Health and Science University, Portland, OR, USA
^3^School of Dentistry, University of Birmingham, Birmingham, UK ^4^Department of Endodontology, School of Dentistry, Oregon Health and Science University, Portland, OR, USA
^5^Center for Regenerative Medicine, Oregon Health and Science University, Portland, OR, USA ^6^Bioengineering Laboratory, Faculty of Dentistry, University of Sydney, Sydney, NSW, Australia. ^#^Authors contributed equally to this work.

^*^Corresponding author

Luiz E. Bertassoni, DDS PhD, e-mail: [bertasso@ohsu.edu.](mailto:bertasso@ohsu.edu)Address: 2730 SW Moody Ave, Collaborative Life Sciences Building, Level 6 6N005, Oregon Health and Science University, Portland, OR, USA. 97201


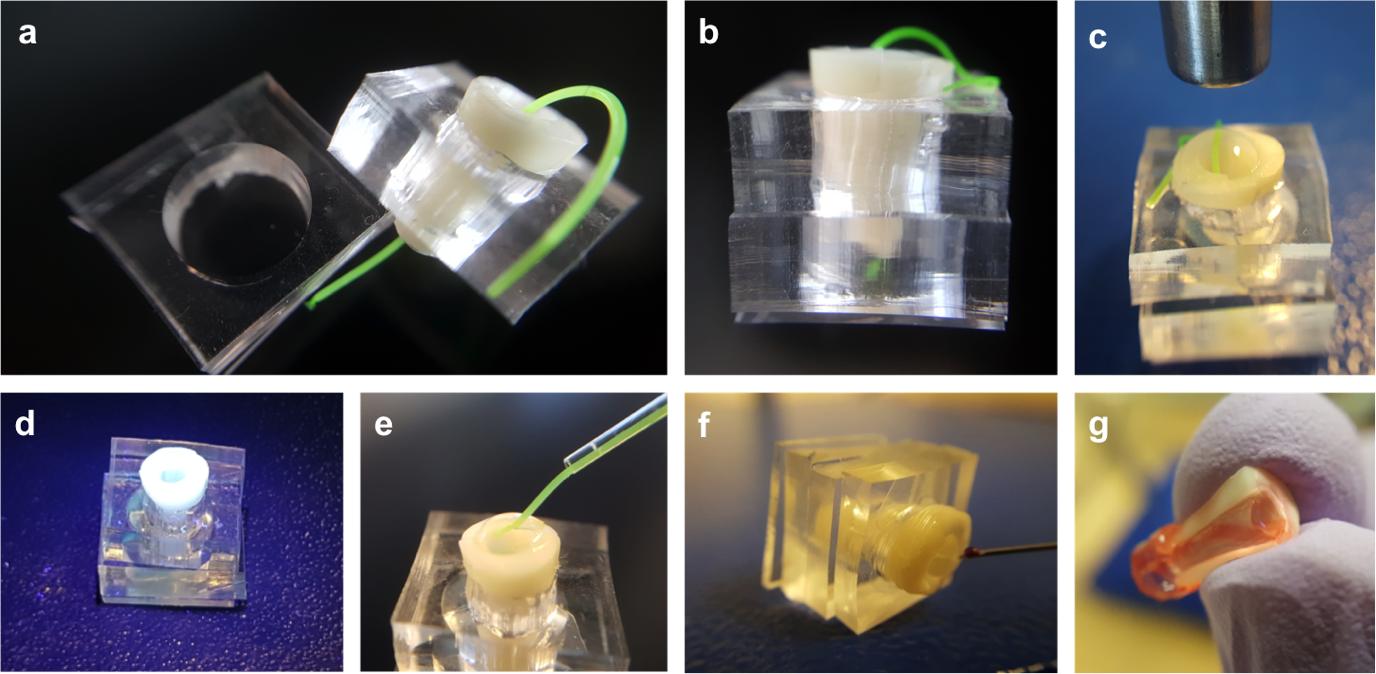


**Supplementary Figure S1:** Fabrication of a pre-vascularized dental pulp-like tissue construct using GelMA hydrogels in the root canal of an endodontically prepared human premolar*. a-b) The prepared root fragments were enclosed in laboratory film and PDMS prior to the placement of a pre-solidified 6% agarose hydrogel fiber (green). c) The tooth was filled with the appropriate volume of GelMA through the cervical opening to reach the predetermined thickness for photopolymerization (3 mm). The filled construct was positioned under the UV light source. d) UV crosslinking of the hydrogel was performed for 30 s per 3 mm layer of hydrogel. e) Agarose hydrogel fibers were removed from the construct by aspirating them with a light vacuum, which created a microchannel. f) ECFCs were seeded into the channels with the constructs positioned horizontally. g) Red dye application illustrated the formation of the channel along the length of the construct. *Teeth were extracted for orthodontic reasons following protocols approved by the institutional review board (IRB) guidelines on research ethics. Patient consent was deemed unnecessary as teeth/patients were not identified, and hence were not considered human subjects research by IRB.


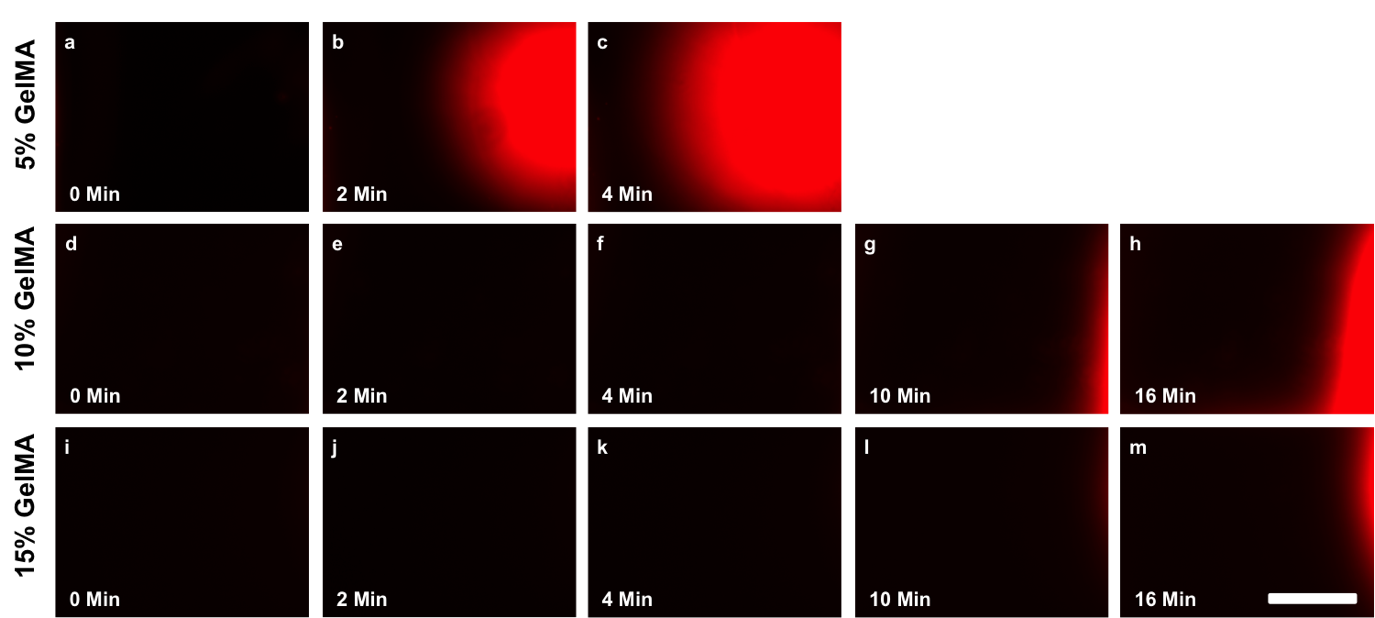


**Supplementary Figure S2:** Permeability of GelMA hydrogels to diffusion of Rhodamine B. GelMA hydrogel in 5 mm samples were exposed to 100 µM Rhodamine B (Sigma) via a channel. The differences in fluorescent diffusion were qualitatively observed under a microscope. The permeability of the hydrogels decreased with increased crosslink density and the diffusion of the Rhodamine B dye was found to be fastest in the 5% (w/v) GelMA hydrogel and slowest in the heavily crosslinked 15% (w/v) GelMA hydrogels. Scale bar represents 1000 µm.

**
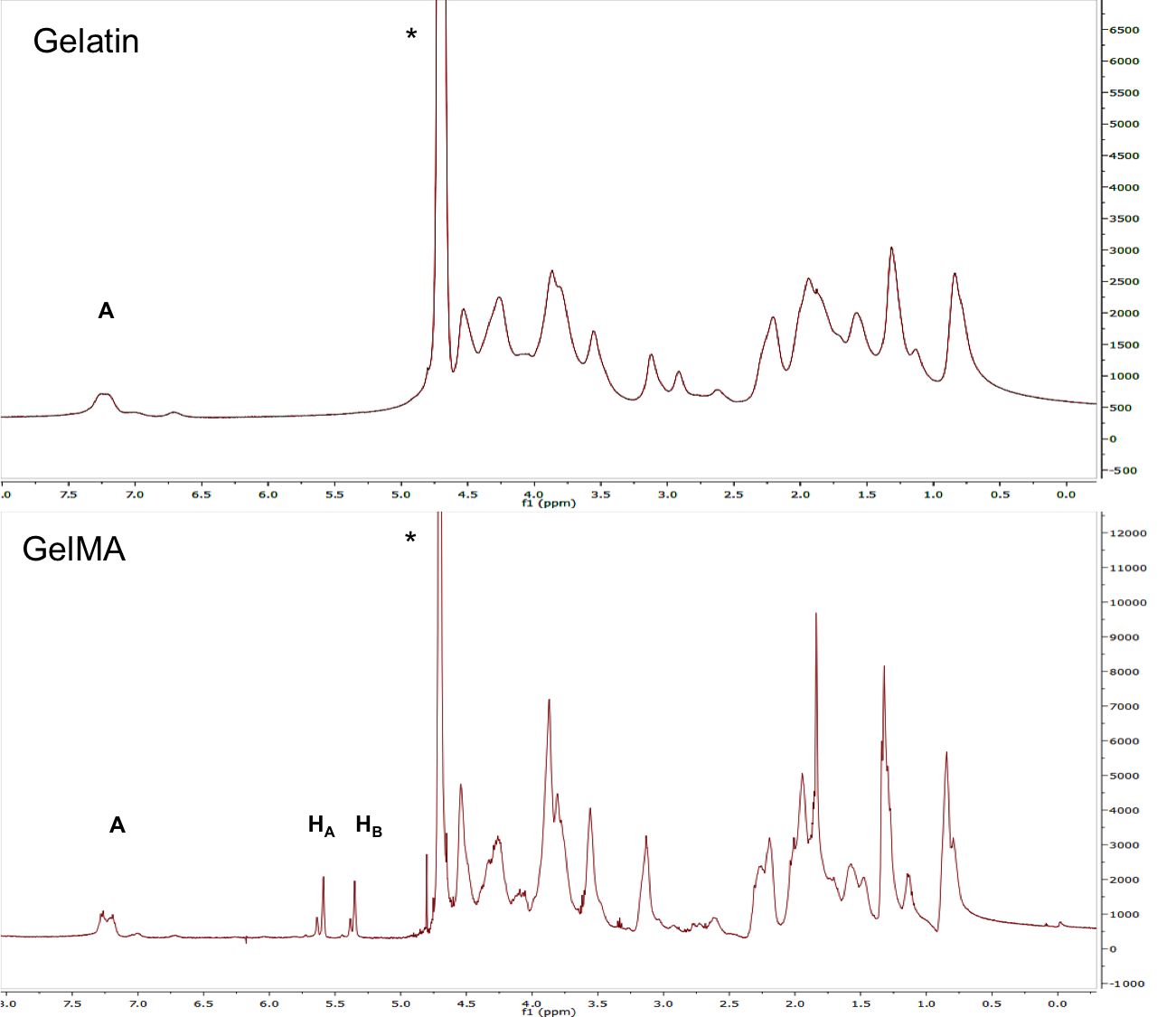
**

**Supplementary Figure S3:** ^1^H-NMR spectra for gelatin (top) and (bottom) gelatin methacryloyl (GelMA). The percentage of methacrylate groups incorporated was determined by comparing the integrated intensity of the vinyl double bond peak (H_A_ and H_B_) to that of the aromatic side chains (A).


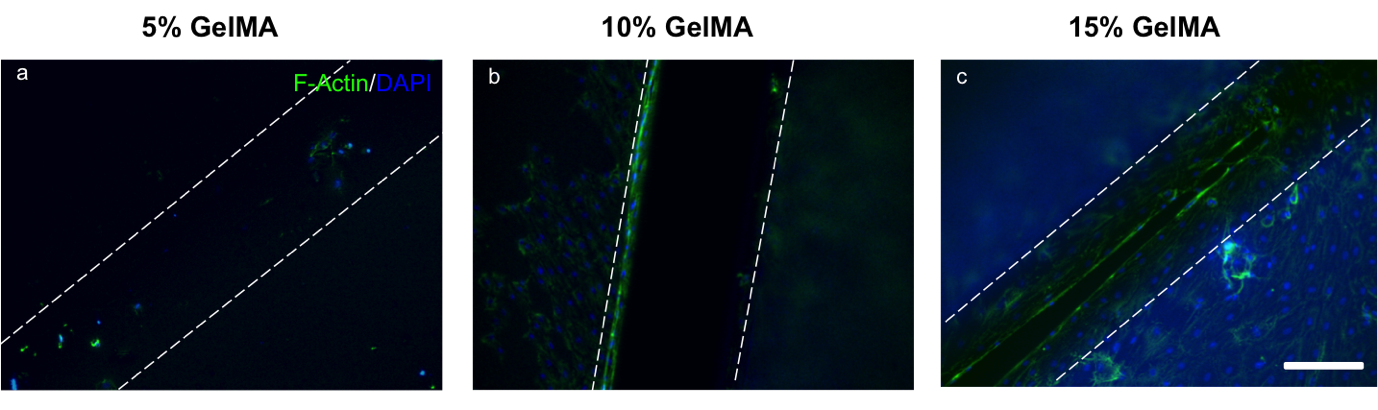


**Supplementary Figure S4:** Representative fluorescent microscope images of endothelial monolayers formed in microchannels within GelMA hydrogels after 5 days. Hydrogels were stained for actin (green) and DAPI (blue) to show cell morphology. (a) 5% hydrogels showed limited cell attachment and monolayer formation. (b) 10% hydrogels, on the other hand, had visible attachment of endothelial cells to the channel walls, (c) and 15% had more discernible, yet incomplete, monolayer formation. Endothelial cells can also be seen in the hydrogels surrounding the monolayer in 10% and 15%, but not in 5% hydrogels.


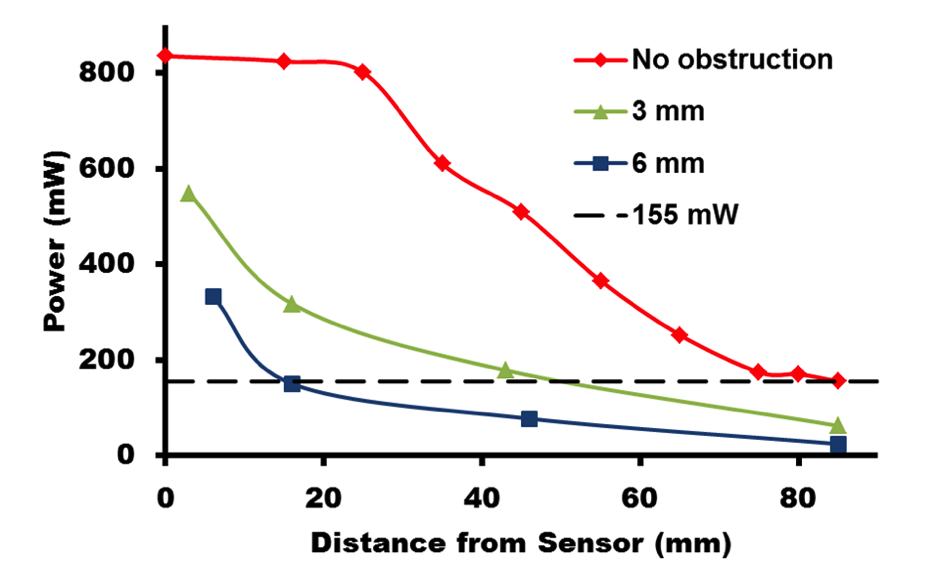


**Supplementary Figure S5:** UV light power relative to the distance from a light power meter. The graph illustrates the intensity of light against a substrate (like a hydrogel) with no obstruction, through a 3 mm long and 3.5 mm wide channel (3 mm), or through a 6 mm long 3.5 mm wide channel (6 mm) inside a dark rubber ring. This approximates the conditions at which Photopolymerization would happen in the oral cavity with the influence of the tooth structure, soft tissues and alveolar bone surrounding the tooth. For comparison, a hydrogel sample subjected to photopolymerization through a 6 mm long channel and 3.5 mm wide opening (i.e. first layer of injected gel in a tooth) will be exposed to 155 mW when the light source is 16 mm away from the sample. A hydrogel subjected to photopolymerization through a 3 mm long channel and 3.5 mm wide opening (i.e. second layer of injected gel in a tooth) will be exposed to 155 mW when the light source is 43 mm away from the sample. A hydrogel subjected to photopolymerization with no obstruction (i.e. third and last layer of an injected hydrogel in a tooth) will be exposed to 155 mW when the light source is 85 mm away from the sample.


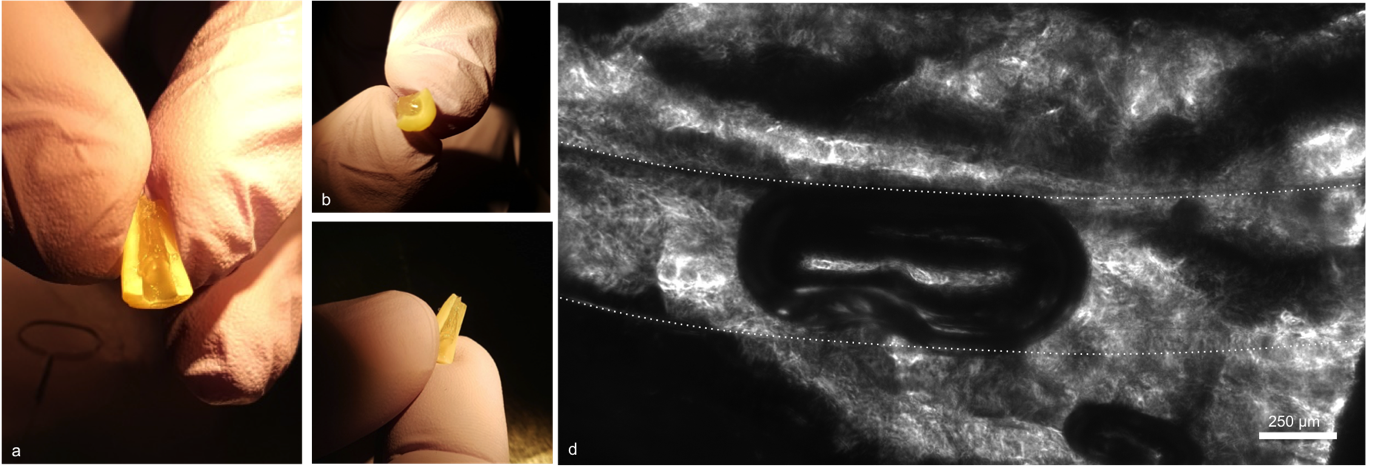


**Supplementary Figure S6:** Cell laden hydrogel tissue construct after 7 days in culture. (a-c) Photographs of intact, cell laden dental pulp-like tissue construct, retrieved from root fragments after 7 days in culture. (d) Phase contrast image of pre-vascularized cell-laden GelMA hydrogel (bubble shows the presence of microchannel) showing the densely cellularized structures both inside and around the microchannel.

**Supplementary Video 1:** Z-stack rendering of OD21 and ECFCs cultured in GelMA hydrogels in the full-length dental pulp-like tissue constructs. OD21 cells appear more spread near the dentin walls, whereas ECFCs are seen coating the microchannel and sprouting into the gel matrix. Cells were stained for actin (green), DAPI (blue) and CD31 (red) on day 7.

**Supplementary Video 2:** Z-stack rendering of ECFCs cultured coating the microchannel and sprouting into the gel matrix. Cells were stained for actin (green), DAPI (blue) and CD31 (red) on day 7.

**Supplementary Video 3:** Video of microchannel fabrication in collagen hydrogels.
